# Effects of Green Tea Polyphenol Epigallocatechin-3-Gallate on Markers of Inflammation and Fibrosis in a Rat Model of Pulmonary Silicosis

**DOI:** 10.3390/ijms24031857

**Published:** 2023-01-17

**Authors:** Jana Adamcakova, Sona Balentova, Romana Barosova, Juliana Hanusrichterova, Pavol Mikolka, Kristian Prso, Juraj Mokry, Zuzana Tatarkova, Dagmar Kalenska, Daniela Mokra

**Affiliations:** 1 Department of Physiology, Jessenius Faculty of Medicine in Martin, Comenius University in Bratislava, 036 01 Martin, Slovakia; 2 Department of Histology and Embryology, Jessenius Faculty of Medicine in Martin, Comenius University in Bratislava, 036 01 Martin, Slovakia; 3 Biomedical Center Martin, Jessenius Faculty of Medicine in Martin, Comenius University in Bratislava, 036 01 Martin, Slovakia; 4 Department of Pharmacology, Jessenius Faculty of Medicine in Martin, Comenius University in Bratislava, 036 01 Martin, Slovakia; 5 Department of Medical Biochemistry, Jessenius Faculty of Medicine in Martin, Comenius University in Bratislava, 036 01 Martin, Slovakia; 6 Department of Anatomy, Jessenius Faculty of Medicine in Martin, Comenius University in Bratislava, 036 01 Martin, Slovakia

**Keywords:** pulmonary silicosis, silica, inflammation, oxidative stress, fibrosis, green tea, polyphenol, epigallocatechin-3-gallate, animal model

## Abstract

Inhalation of silica particles causes inflammatory changes leading to fibrotizing silicosis. Considering a lack of effective therapy, and a growing information on the wide actions of green tea polyphenols, particularly epigallocatechin-3-gallate (EGCG), the aim of this study was to evaluate the early effects of EGCG on markers of inflammation and lung fibrosis in silicotic rats. The silicosis model was induced by a single transoral intratracheal instillation of silica (50 mg/mL/animal), while controls received an equivalent volume of saline. The treatment with intraperitoneal EGCG (20 mg/kg, or saline in controls) was initiated the next day after silica instillation and was given twice a week. Animals were euthanized 14 or 28 days after the treatment onset, and the total and differential counts of leukocytes in the blood and bronchoalveolar lavage fluid (BALF), wet/dry lung weight ratio, and markers of inflammation, oxidative stress, and fibrosis in the lung were determined. The presence of collagen and smooth muscle mass in the walls of bronchioles and lung vessels was investigated immunohistochemically. Early treatment with EGCG showed some potential to alleviate inflammation, and a trend to decrease oxidative stress-induced changes, including apoptosis, and a prevention of fibrotic changes in the bronchioles and pulmonary vessels. However, further investigations should be undertaken to elucidate the effects of EGCG in the lung silicosis model in more detail. In addition, because of insufficient data from EGCG delivery in silicosis, the positive and eventual adverse effects of this herbal compound should be carefully studied before any preventive use or therapy with EGCG may be recommended.

## 1. Introduction

Pulmonary silicosis is an incurable fibrotizing disease caused by a prolonged and/or a long-term exposition to silica particles [1]. Inhaled silica causes an accumulation of cells (macrophages, neutrophils, lymphocytes and others) in the lung interstitium, thickening of the interstitium mass, formation of hyalinized fibrotic nodules, and deposition of collagen fibers. Although the interactions among the pathomechanisms have not been completely elucidated yet, the above-mentioned changes are likely associated with persistent silica-induced inflammation and oxidative stress [2,3,4,5]. 

Due to their piezoelectric properties, silica crystals are directly toxic to the lung tissue as they trigger a generation of reactive oxygen species (ROS) on their surface [2,6]. Inhaled silica is rapidly recognized by the surface receptors of airway epithelial cells that triggers a complex immune response, mediated particularly by activated macrophages, dendritic cells, and lymphocytes [7,8]. Alveolar macrophages envelop the silica particles [2,9]; however, the internalized silica cannot be destroyed by lysosomal enzymes, which leads to damage of the lysosomes and a release of enzymes and silica into the lung environment. Silica freed from disabled macrophages can be engulfed by other macrophages, generating a vitious cycle of the lung tissue injury [2]. Activation of the surface receptors, generation of ROS, and released lysosomal enzymes, including protease cathepsin B, result in activation of the related pro-inflammatory pathways, e.g., nuclear factor (NF)-κB and mitogen-activated protein kinase (MAPK), etc. These may also stimulate an activation of the nucleotide-binding and oligomerization domain-like receptor (NLR)P3 inflammasome [4,10]. NLRP3 inflammasome is a special intracellular receptor complex responsible for the activation of caspase-1 inducing a pyroptosis, a pro-inflammatory type of cell death associated with a release of interleukin (IL)-1α, IL-1β, IL-18, high mobility group box 1 protein (HMGB1), ROS etc. [11]. These substances provoke an expression of additional pro-inflammatory cytokines and chemokines, including tumor necrosis factor (TNF)α which prompts the influx of neutrophils into the lung and causes damage to the cells. The above-mentioned bioactive substances enhance recruitment of fibroblasts and induce expression of pro-fibrotic transforming growth factor (TGF)-β which causes activation, proliferation, and transdifferentiation of epithelial cells and fibroblasts into myofibroblasts producing components of the extracellular matrix, e.g., collagen. Overproduction of pro-fibrotic substances and recruitment of collagen- and fibronectin-generating cells results in the creation of silicotic nodules, scarring the lung tissue, and the diminution of areas supplying a gas exchange [12,13].

Complex pathomechanisms of the disease, the persistence of silica particles in the lung, and the perpetuation of pro-inflammatory and pro-fibrotic cascades means that there has been no causal treatment of silicosis. A majority of patients are treated only symptomatically using bronchodilators and antitussic and mucolytic drugs, and patients are recommended to avoid additional exposures to silica [1,14]. Nevertheless, considering the role of silica-induced inflammation in the pathophysiology of silicosis, the testing of novel approaches targeting inflammation and fibrosis, especially in the early stages of the disease, is particularly important. As reviewed in our article [5], promising results have been published for treatment with, for example, antifibrotic agents approved for idiopathic pulmonary fibrosis pirfenidone [15] and nintedanib [16], antioxidant N-acetylcysteine [17,18], agents blocking pro-inflammatory cytokines [19,20], inhibitors of phosphodiesterases increasing cyclic adenosine monophosphate (cAMP) or guanosine monophosphate (cGMP) [21,22], or corticosteroids [21,23].

In addition to the previously mentioned approaches, there are a number of herbal drugs that can influence one or even several pathomechanisms of silicosis and, thereby, can mitigate the silica-induced inflammation and/or fibrosis [24]. These include sodium tanshinone IIA sulfonate [25], kaempferol [26], astragaloside IV [27], dioscin [28], oleanolic acid [29], hesperetin [30], or emodin [31]. Among them, polyphenols from a green tea plant (*Camellia sinensis*) take an exceptional place, particularly (–)-epigallocatechin-3-gallate (EGCG). EGCG possesses a wide variety of anti-inflammatory, anti-fibrotic, anti-tumorous, and metabolic effects via the modulation of many signaling cascades. Its therapeutic potential has been demonstrated for cancer [32,33], neurological disorders [34,35], cardiovascular diseases [36,37], and metabolic disorders, including obesity and diabetes mellitus [38,39]. In addition, as published in our recent article [40], administration of EGCG may be of benefit in respiratory diseases with inflammatory, oxidative, and fibrotizing processes in the pathogenesis. In experimental models of lung fibrosis induced by bleomycin [41,42,43], irradiation [44], cyclophosphamide [45] or paraquat [46], EGCG exerted significant anti-inflammatory, antioxidant and anti-fibrotic effects. In addition, favorable results have been recently demonstrated for EGCG-encapsulated particles in the rat model of lung silicosis [47]. Considering the above-mentioned facts, this study aimed to determine the efficacy of EGCG on early inflammatory and fibrotic changes in the lungs of silica-instilled rats, in an effort to evaluate its future perspectives in the treatment of lung silicosis. To reduce a biological degradation of EGCG by the gastrointestinal system [40], intraperitoneal administration of EGCG was preferred in accordance with the above-mentioned authors [41,42,43,44].

## 2. Results

### 2.1. Changes in the Counts of Leukocytes in the Blood and Bronchoalveolar Lavage Fluid (BALF)

The total count of leukocytes in the blood was significantly elevated in the silica-instilled animals compared to the saline-instilled controls after 14 days (*p* < 0.05), while the increase was non-significant after 28 days (*p* > 0.05). Although no differences were observed in the percentages of the individual types of leukocytes in silica-instilled animals vs. controls, the analysis of absolute counts revealed that the above-mentioned changes were attributable to increases in absolute counts of circulating lymphocytes (*p* < 0.05 after 14 days, *p* > 0.05 after 28 days), monocytes (*p* > 0.05 after 14 days, *p* < 0.01 after 28 days), and eosinophils (*p* > 0.05). In the EGCG-treated animals, the total count of leukocytes raised (*p* > 0.05 after 14 days, *p* < 0.05 after 28 days), was associated with increases in circulating neutrophils (*p* < 0.01 after 14 days, *p* < 0.001 after 28 days) and monocytes (*p* > 0.05 after 14 days, *p* < 0.01 after 28 days), in comparison with the non-treated silica-instilled animals (Table 1).

The total count of leukocytes in the BALF was slightly, but non-significantly elevated in the silica-instilled animals compared to the controls (*p* > 0.05). In silica-instilled animals compared to saline-instilled controls, increases in percentages and absolute counts of neutrophils (both *p* < 0.001 after 14 days, both *p* < 0.001 after 28 days), in percentages (*p* < 0.01 after 14 days, *p* < 0.001 after 28 days) and absolute counts (both *p* < 0.05 after 14 and 28 days) of eosinophils, and in percentages (*p* < 0.05 after 14 days, *p* < 0.001 after 28 days) and absolute counts (*p* > 0.05 after 14 days, *p* < 0.01 after 28 days) of lymphocytes were found. Contrarily, the percentages of macrophages decreased (both *p* < 0.001 after 14 and 28 days), but the absolute counts of macrophages declined insignificantly (both *p* > 0.05 after 14 and 28 days). Treatment with EGCG slightly, but non-significantly lowered a total count of BALF cells compared to the non-treated silica-instilled animals (*p* > 0.05 after 28 days). In addition, EGCG treatment significantly decreased percentages (*p* < 0.01 after 14 days, *p* < 0.05 after 28 days) and absolute counts (*p* < 0.001 after 14 days, *p* < 0.05 after 28 days) of neutrophils and percentages, and absolute counts of lymphocytes (both *p* < 0.05 after 28 days), while decreases in eosinophils were insignificant (*p* > 0.05 after 14 and 28 days) (Table 2).

### 2.2. Changes in the Oxidant/Antioxidant Markers

**Figure 1:**
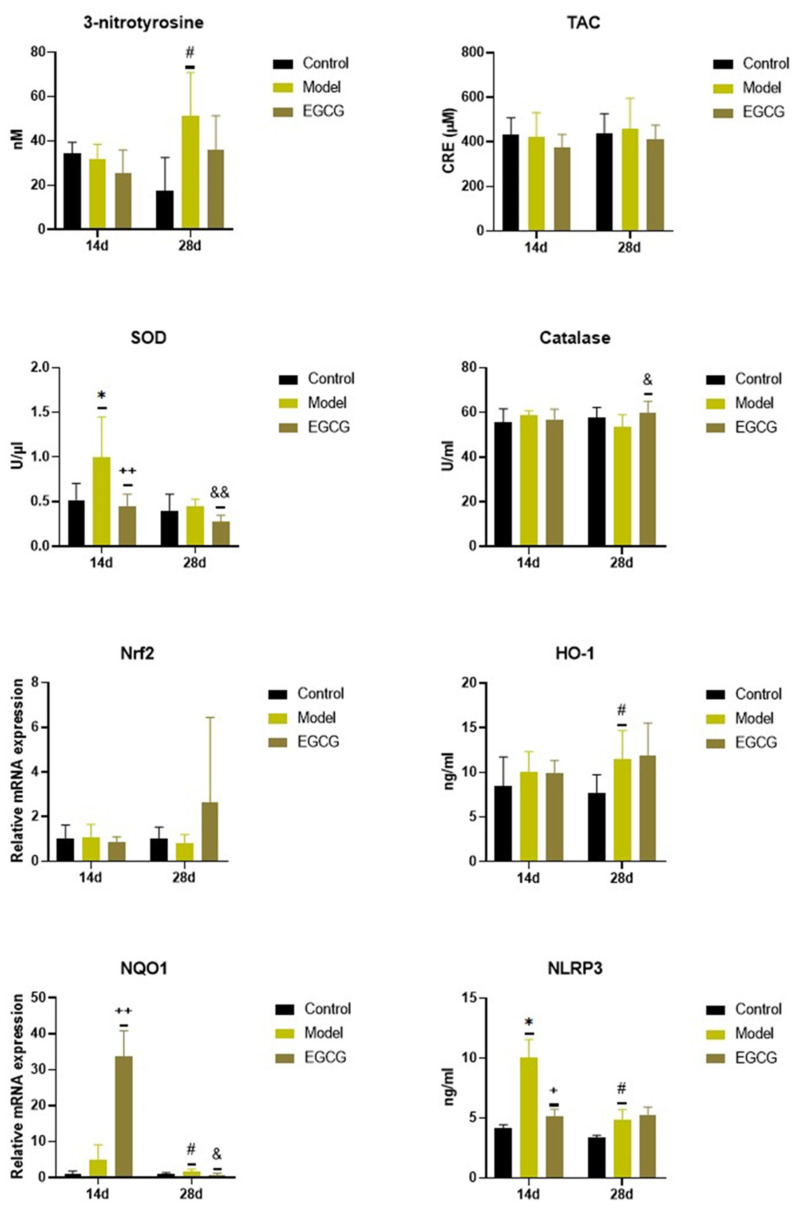
Changes in the oxidant/antioxidant markers in the lung of saline-instilled animals (Control), silica-instilled non-treated animals (Model), and EGCG-treated silica-instilled animals (EGCG) after 14 days (14d) or 28 days (28d) of the treatment delivery. Abbreviations: TAC: total antioxidant capacity; SOD: superoxide dismutase; Nrf2: nuclear factor erythroid-derived 2-like factor 2; HO-1: heme oxygenase; NQO-1: NAD(P)H:quinone oxidoreductase-1; NLRP3: nucleotide-binding and oligomerization domain-like receptor. For statistical differences among the groups: * *p* < 0.05 for Model14d vs. Control14d; + *p* < 0.05 and ++ *p* < 0.01 for EGCG14d vs. Model14d; # *p* < 0.05 for Model28d vs. Control28d; ^&^ *p* < 0.05 and ^&&^ *p* < 0.01 for EGCG28d vs. Model28d.

Instillation of silica caused significant changes in the oxidant/antioxidant markers, while increases in markers of oxidation were accompanied by increases in some antioxidant systems. Concentration of 3-nitrotyrosine, a marker of protein oxidation, did not change after 14 days (*p* > 0.05) but were significantly elevated (*p* < 0.05) after 28 days in the silica-instilled animals compared to controls, while EGCG slightly decreased its level after 28 days compared to the non-treated silica-instilled animals (*p* > 0.05) (Figure 1). 

There were no significant changes in the total antioxidant capacity (TAC) and catalase of the silica-instilled vs. saline-instilled animals (all *p* > 0.05 after 14 and 28 days); however, the concentration of catalase increased after the treatment with EGCG (*p* < 0.05 after 28 days) compared to the non-treated animals (Figure 1). More obvious differences were found for superoxide dismutase (SOD) between the silica-instilled vs. saline-instilled animals (*p* < 0.05 after 14 days, *p* > 0.05 after 28 days) as well as between the EGCG-treated vs. non-treated animals (both *p* < 0.01 after 14 and 28 days (Figure 1).

Despite no obvious differences in mRNA expressions of the antioxidant nuclear factor erythroid-derived 2-like factor 2 (Nrf2) system in the silica-instilled lung compared to controls, increases in relative mRNA expressions of the associated enzymes heme oxygenase (HO)-1 and NAD(P)H:quinone oxidoreductase (NQO)-1 (both *p* > 0.05 after 14 days, *p* < 0.05 after 28 days) were found. EGCG treatment caused an elevated expression of NQO-1 after 14 days (*p* < 0.01), but decreased expression of NQO-1 after 28 days (*p* < 0.05), and an insignificantly increased expression of Nrf2 after 28 days (*p* > 0.05), however it showed no effect on HO-1 (*p* > 0.05) (Figure 1). Concentration of NLRP3, which is closely linked with oxidative stress and inflammation, was significantly elevated in the silica-instilled animals compared to controls (both *p* < 0.05 after 14 and 28 days, while treatment with EGCG lowered NLRP3 concentration after 14 days (*p* < 0.05) (Figure 1). 

### 2.3. Changes in the Inflammatory Markers

**Figure 2:**
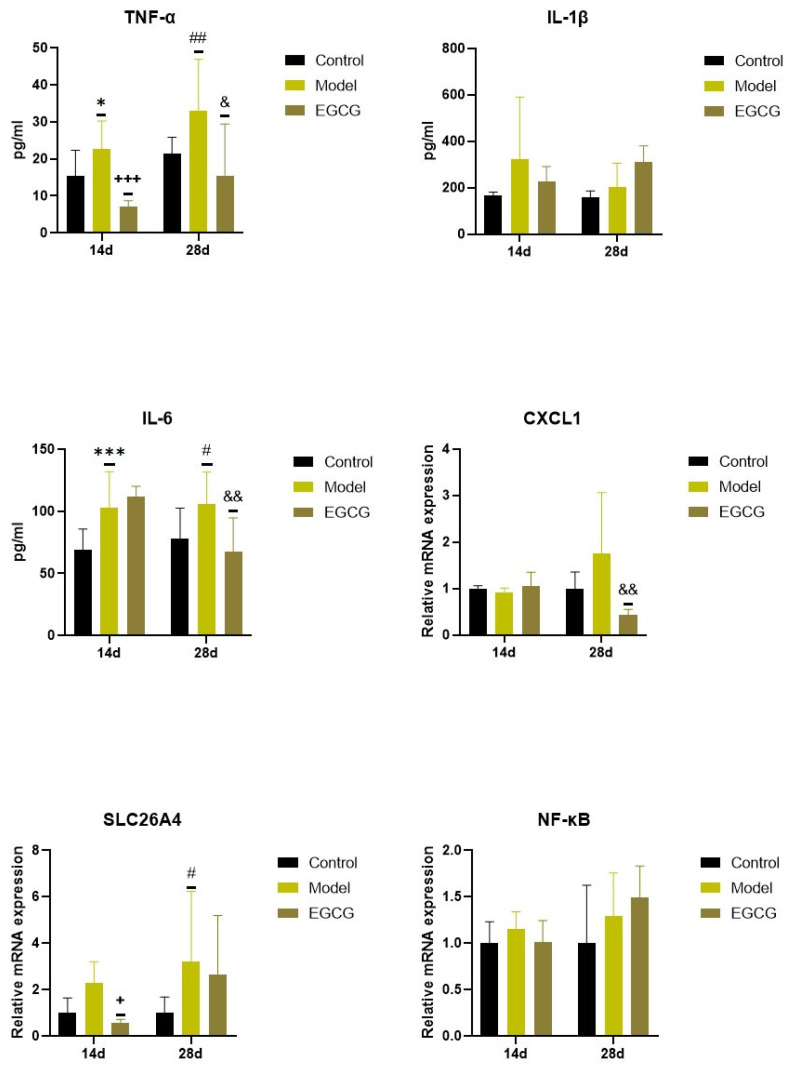
Changes in the inflammatory markers in the lungs of saline-instilled animals (Control), silica-instilled non-treated animals (Model), and EGCG-treated silica-instilled animals (EGCG) after 14 days (14d) or 28 days (28d) of the treatment delivery. Abbreviations: TNFα: tumor necrosis factor alpha; IL-1β: interleukin-1beta; IL-6: interleukin-6; CXCL1: chemokine (C-X-C motif) ligand 1; SLC26A4: solute carrier family 26, member 4; NF-κB: nuclear factor-kappa B. For statistical differences among the groups: * *p* < 0.05 and *** *p* < 0.001 for Model14d vs. Control14d; + *p* < 0.05 and +++ *p* < 0.001 for EGCG14d vs. Model14d; # *p* < 0.05 and ## *p* < 0.01 for Model28d vs. Control28d; ^&^ *p* < 0.05 and ^&&^ *p* < 0.01 for EGCG28d vs. Model28d.

Concentrations of pro-inflammatory cytokines increased in the silica-instilled lung compared to the controls (for TNFα: *p* < 0.05 after 14 days, *p* < 0.01 after 28 days; for IL-1β: both *p* > 0.05; for IL-6: *p* < 0.001 after 14 days, *p* < 0.05 after 28 days), despite mRNA expression of NF-κB, it was elevated only insignificantly (both *p* > 0.05) (Figure 2). 

Increases in two additional inflammatory markers, chemokine (C-X-C motif) ligand 1 (CXCL1) and solute carrier family 26, member 4 (SLC26A4), were found in the silica-instilled animals compared to controls after 28 days (*p* > 0.05 for CXCL1, *p* < 0.05 for SLC26A4) (Figure 2). Treatment with EGCG decreased lung concentrations of TNFα (*p* < 0.001 after 14 days, *p* < 0.05 after 28 days), IL-6 (*p* < 0.01 after 28 days), and CXCL1 (*p* < 0.01 after 28 days), but showed no decrease in other markers (IL-1β, SLC26A4, NF-κB) (Figure 2).

### 2.4. Changes in the Wet/Dry (W/D) Lung Weight Ratio

**Figure 3:**
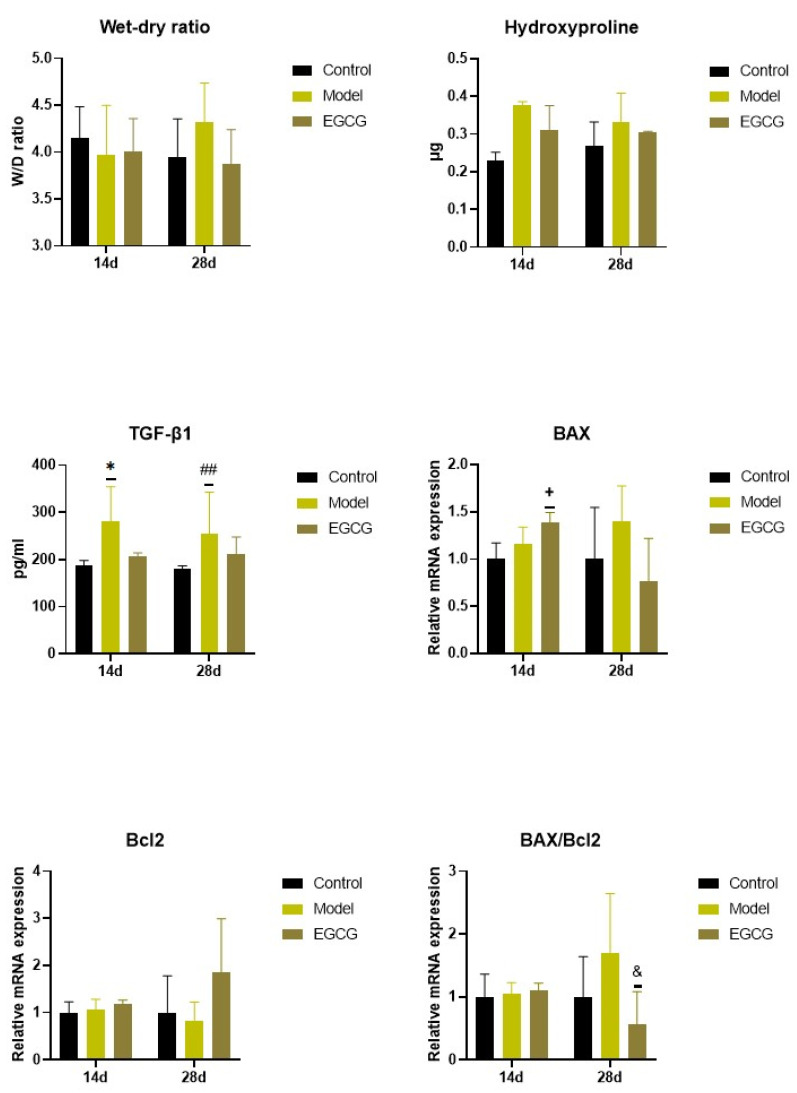
Changes in the W/D ratio and in the markers of apoptosis and fibrosis in the lungs of saline-instilled animals (Control), silica-instilled non-treated animals (Model), and EGCG-treated silica-instilled animals (EGCG) after 14 days (14d) or 28 days (28d) of the treatment delivery. Abbreviations: TGF-β1: transforming growth factor-beta 1; Bcl-2: B-cell lymphoma 2 protein; BAX: Bcl-2 associated X-protein. For statistical differences among the groups: * *p* < 0.05 for Model14d vs. Control14d; + *p* < 0.05 for EGCG14d vs. Model14d; ## *p* < 0.01 for Model28d vs. Control28d; ^&^ *p* < 0.05 for EGCG28d vs. Model28d.

Except for an insignificant increase in W/D ratio between the non-treated silica-instilled animals and saline-instilled controls after 28 days, analysis of the W/D ratio, a marker of lung edema formation, showed no differences among the groups (all *p* > 0.05) (Figure 3).

### 2.5. Changes in the Lung Cell Apoptosis

The PCR analysis showed that relative expressions of anti-apoptotic B-cell lymphoma 2 protein (Bcl-2) remained without obvious changes (both *p* > 0.05 after 14 and 28 days), while expression of pro-apoptotic Bcl-2 associated X-protein (Bax) and Bax/Bcl-2 ratio slightly, but non-significantly increased in the lungs of the silica-instilled animals compared to controls (both *p* > 0.05 after 14 and 28 days). EGCG treatment caused increases in both Bax (*p* < 0.05) and Bcl-2 (*p* > 0.05) after 14 days, while Bax decreased and Bcl-2 increased after 28 days that resulted in a decline in Bax/Bcl-2 ratio (*p* < 0.05) compared to the non-treated silica-instilled animals (Figure 3).

### 2.6. Changes of Markers of Lung Fibrosis

Concentrations of hydroxyproline, a degradation product of collagen, was slightly elevated in the silica-instilled animals compared to controls, and the EGCG treatment caused a modest decline compared to the non-treated animals; however, all these changes were non-significant (all *p* > 0.05) (Figure 3). 

Levels of TGF-β1, a principal regulator of the lung fibrosis, significantly increased in the lung tissue in the silica-instilled animals compared to saline-instilled controls (*p* < 0.05 after 14 days, *p* < 0.01 after 28 days), while EGCG treatment caused a small but insignificant decrease (*p* > 0.05 after 14 and 28 days) (Figure 3).

### 2.7. Immunohistochemical Detection of Collagen and α-Smooth Muscle Actin (α-SMA) in the Lung

**Figure 4:**
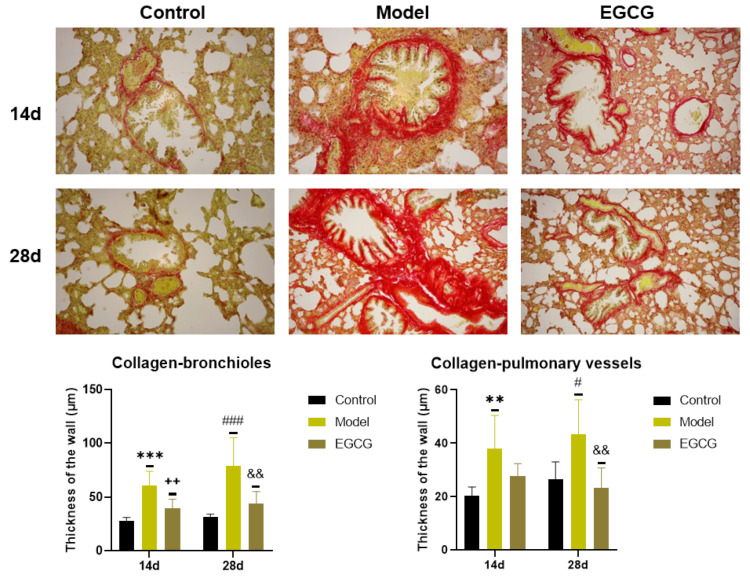
Immunohistochemical determination of the collagen presence in the lung of saline-instilled animals (Control), silica-instilled non-treated animals (Model), and EGCG-treated silica-instilled animals (EGCG) after 14 days (14d) or 28 days (28d) of the treatment delivery. Presence of collagen was verified by using Sirius red staining and was expressed as a thickness of the walls of bronchioli and pulmonary vessels (in μm). For statistical differences among the groups: ** *p* < 0.01 and *** *p* < 0.001 for Model14d vs. Control14d; ++ *p* < 0.01 for EGCG14d vs. Model14d; # *p* < 0.05 and ### *p* < 0.001 for Model28d vs. Control28d; ^&&^ *p* < 0.01 for EGCG28d vs. Model28d.

Immunohistochemical investigation by Sirius red staining was used for the detection of collagen accumulation. The results showed a significant increase in the thickness of the walls of bronchioles (both *p* < 0.001 after 14 and 28 days) and pulmonary vessels (*p* < 0.01 after 14 days, *p* < 0.05 after 28 days) in the silica-instilled vs. saline-instilled animals. In EGCG-treated animals, a decrease in collagen mass was observed in the walls of bronchioles (both *p* < 0.01 after 14 and 28 days) and pulmonary vessels (*p* > 0.05 after 14 days, *p* < 0.01 after 28 days) (Figure 4).

**Figure 5:**
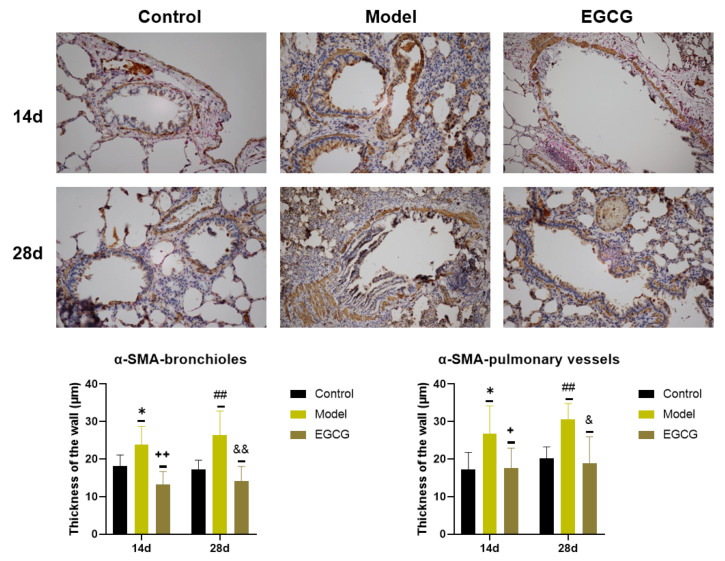
Immunohistochemical determination of the smooth muscle mass presence in the lungs of saline-instilled animals (Control), silica-instilled non-treated animals (Model), and EGCG-treated silica-instilled animals (EGCG) after 14 days (14d) or 28 days (28d) of the treatment delivery. Presence of smooth muscle mass was verified by using antibody against α-smooth muscle actin (SMA) and was expressed as a thickness of the walls of bronchioli and pulmonary vessels (in μm). For statistical differences among the groups: * *p* < 0.05 for Model14d vs. Control14d; + *p* < 0.05 and ++ *p* < 0.01 for EGCG14d vs. Model14d; ## *p* < 0.01 for Model28d vs. Control28d; ^&^ *p* < 0.05 and ^&&^ *p* < 0.01 for EGCG28d vs. Model28d.

Detection of α-SMA, a marker of connective tissue accumulation, was performed using a specific α-SMA antibody. We found that the thicknesses of the bronchiolar walls (*p* < 0.05 after 14 days, *p* < 0.01 after 28 days) and pulmonary vessels walls (*p* < 0.05 after 14 days, *p* < 0.01 after 28 days) significantly elevated in the silica-instilled animals compared to the saline-instilled animals. Treatment with EGCG decreased a generation of the smooth muscle mass in the bronchioles (both *p* < 0.01 after 14 and 28 days) and pulmonary vessels (both *p* < 0.05 after 14 and 28 days) (Figure 5).

Summary of the EGCG effects on measured markers is provided in Table 3.

## 3. Discussion

Inhaled silica triggers complex cellular responses, leading to the activation of inflammatory and pro-fibrotic processes. In this study, the orotracheal instillation of silica caused a mobilization of inflammatory cells that was associated with their activation, and the increased production of pro-inflammatory cytokines and other bioactive substances. Hand-in-hand with inflammation, enhanced fibrogenic activity led to the increased generation of collagen and smooth muscle mass in the walls of bronchioles and pulmonary vessels. Early treatment with natural flavonoid EGCG mitigated a severity of inflammation and partially prevented fibrotic changes in the lung tissue, suggesting a therapeutic potential of EGCG in the alleviation of silica-induced inflammatory and fibrotic lung injury.

Soon after inhalation into the airways, silica affects the airway epithelial cells which participate in the immune response against the penetrating particles [8]. Particle-induced activation of receptors on the airway epithelial cells triggers a production of pro-inflammatory mediators that subsequently activates the macrophages, dendritic cells and innate lymphoid cells. The presentation of inhaled antigen on the surface of these antigen-presenting cells to different subsets of T helper cells results in the activation of various immune cells including lymphocytes and granulocytes, mainly neutrophils, of which recruitment is a typical sign of acute inflammation [7,8]. Activated cells release a broad array of inflammatory mediators such as cytokines, ROS, and reactive nitrogen species (RNS) which pronounce a recruitment of inflammatory cells into the lung [48]. In this study, both absolute and relative counts of leukocytes are provided to show a complex dynamic of the individual types of cells that may have a different rate for migration into the tissue, or for replenishment from the pools or by production in the bone marrow. In the event that the counts of several types of leukocytes change simultaneously in a different extent, as it is observed in silicosis, absolute values seem to be more precise markers of cell count changes. Thus, a total number of leukocytes and counts of monocytes and lymphocytes in the blood increased in the silica-injured animals compared to the saline-instilled controls. This suggests a mobilization of these cells from the reserves, or a stimulated production in the bone marrow after the silica trigger. In the BALF of silica-instilled animals, the total count of cells was slightly elevated, and lymphocytes, together with neutrophils and eosinophils, were found in higher percentages and absolute counts than in the controls. In agreement with our results, lymphocytes were abundant in other silica-instilled models where the significant accumulation of lymphocytes was detected in the alveolar compartment, in lung parenchymal lesions and nodules, but also in extended bronchial-associated lymphoid tissues and thoracic lymph nodes [22,49,50,51]. Similarly, elevated total counts of cells, as well as increased counts of phagocytes, particularly neutrophils and macrophages, in the BALF were demonstrated by other authors [22,51,52,53,54]. Higher migration of immune cells into the lung in our study can partially result from elevated concentrations of chemokine CXCL1, which acts as a chemokine for neutrophils and other cells at the site of injury, and contributes to the regulation of the inflammatory response. Comparable findings were also demonstrated in in vitro exposure of bronchial cells to silica [55], as well as in silica-instilled mice [23]. Although the numbers of circulating monocytes in our study were elevated, and an intensive migration from circulation into the injured lung was expected, the percentage and absolute count of macrophages in the BALF decreased compared to the controls. Similarly, Porter et al. [56] found that after 16 resp. 30 days of the daily aerosolized silica exposure (i.e., in comparable time relations to our experiment), there was a significant increase in the total count of polymorphonuclears in BALF and a slight increase in the blood neutrophil count, but no differences in alveolar macrophages in BALF nor in blood monocytes compared to the controls. We can hypothesize that monocytes transforming to macrophages in the lung may be destroyed early on when in contact with highly toxic silica [2]. These cells may also move to the lung interstitium, where they are unavailable for the bronchoalveolar lavage procedure, and where they continue in repetitive but unsuccessful destruction of ingested silica particles and thereby contribute to the progression of silica-induced lung injury [57,58]. 

A finding of slightly elevated counts of BALF cells (i.e., total count, macrophages, and neutrophils) after 28 days vs. 14 days in the saline-instilled controls is of interest, suggesting some local non-specific inflammatory changes in the lung due to saline administration. Similar temporary non-significant changes in several markers of inflammation and fibrosis were also demonstrated by other authors [59,60,61]. Luo et al. [61] hypothesized that these changes may be associated with a short-term activation of macrophages.

As mentioned before, silica-induced generation of ROS and RNS, and the subsequent oxidative modifications of biomolecules participate in the silica-associated lung injury and fibrosis [62]. However, hand-in-hand with an increase in the production of oxidants is also the production of anti-oxidants. This is a compensatory response to the silica-induced oxidant/antioxidant dysbalance increases that may result in discrepancies in the results obtained from various studies, due to different time relations of oxidant production vs. antioxidant depletion. For instance, we found that 3-nitrotyrosine, a sensitive marker of protein nitration, was significantly elevated in the lung homogenate after 28 days of silica exposure compared to healthy controls, while simultaneously an increase in SOD concentration was found, but with no changes in TAC or catalase. In accordance with our results, markers of both oxidant and antioxidant activities increased after intranasal silica exposure in rats [53]; however, in other rat models of intranasally-provoked silicosis, a significant rise in malonyldialdehyde and nitrite/nitrate content in the lung was associated with a decline in lung glutathione content and SOD activity [22]. Additionally, this study evaluated the role of Nrf2 signaling and its regulated antioxidant enzymes HO-1 and NQO-1 in maintaining redox homeostasis. Although no differences in Nrf2 mRNA expression were found either after 14 or 28 days, significant increases in the silica-instilled animals vs. controls after 28 days were found for both HO-1 and NQO-1. HO-1 is a rate-limiting enzyme in heme catabolism, and its production likely increases due to elevated ROS-induced activation of extracellular signal-regulated kinases (ERK). HO-1 enhances heme degradation to carbon monoxide, free iron, and bilirubin. Bilirubin scavenges ROS, and bilirubin together with carbon monoxide attenuates the silica-induced ERK activation, leading to reduction of the silicosis progression [63]. In this study, the concentration of HO-1 elevated after 14 days, but particularly after 28 days of silica instillation in comparison to saline-instilled animals. Similar to our results, increased serum levels and pulmonary mRNA expression of HO-1 were demonstrated both in patients with silicosis and silica-instilled mice [64]. Contrarily, in Sprague-Dawley rats, after 56 days of single intranasal instillation of silica, lower HO-1 and Nrf2 was found compared to controls [53]. 

Activation of the Nrf2 antioxidant system may also reduce the oxidative stress-induced apoptosis of the lung cells [62,65,66,67]. In this study, mRNA expressions of cell apoptosis regulators, proteins Bax and Bcl-2, were measured. We found that expression of pro-apoptotic protein Bax slightly increased and expression of anti-apoptotic protein Bcl-2 decreased in the silica-instilled animals compared to controls, which was associated with a slightly increased Bax/Bcl-2 ratio. Similar results in response to silica were also demonstrated by other authors [68,69].

The activation of immune cells due to the persistence of silica in the lung leads to activation of pro-inflammatory transcription factors such as NF-κB, activation of NLRP3 inflammasome, and associated production of cytokines and other bioactive substances [4,6,70]. In our study, higher concentrations of TNFα, IL-1β and IL-6 as well as NLRP3 and NF-κB were detected in the lung homogenates of the silica-instilled animals compared to healthy controls. A finding of elevated concentrations of pro-inflammatory cytokines is in agreement with other studies, where higher concentrations of TNFα were found in BALF or in the lungs of the silica-instilled animals [22,53], while a production of IL-1 and TNFα in the BALF cells of aerosolized-silica exposed rats gradually increased and reached significant differences in comparison to the healthy control at 10 or 30 days of silica daily exposure [56]. Activation of NLRP3 inflammasome was previously confirmed in the cultures of bronchial epithelial cells and in the macrophages in response to crystalline silica, but also in the in vivo model [71,72]. However, silica-induced lung inflammation likely goes hand-in-hand with stimulation of anti-inflammatory mechanisms. Paradoxically, there is a growing body of evidence from animal experiments suggesting that anti-inflammatory cytokines such as IL-10 and TGF-β (which also acts as a potent fibrogenic agent) may also exert a detrimental activity during the establishment of lung fibrosis [48]. Finally, PCR analysis of SLC26A4 (or pendrin) showed an increase in its relative mRNA expression in the silica-injured animals compared to controls after 14 days, and this difference became significant after 28 days of silica exposure. Pendrin, as an anion transporter located at the apical side of airway epithelial cells, acts as a downstream effector of the IL-4/IL-13 signaling pathway and participates, e.g., in airway inflammation in asthma. Our results confirmed the recent findings of other authors [73,74] suggesting that pendrin may play a role in early silicosis and may serve as a potential early biomarker of silicosis, as well.

Exaggerated accumulation and activation of cells in the lung may result in a higher generation of lung edema fluid. However, no difference in the W/D ratio in our study was found after 14 days, and only a negligible increase was observed after 28 days of silica exposure compared to controls. Contrarily, a significant increase in the total protein content in BALF was found in intranasal silica-exposed Sprague-Dawley rats compared to healthy controls; however, the duration of that study was 8 weeks [22]. In the same strain of rats, a single intranasal instillation of silica increased the W/D ratio and total protein level in BALF, but this was measured after 56 days of silica exposure [53]. 

In fibrotic diseases including pulmonary silicosis, a net accumulation of extracellular matrix proteins in affected organs leads to their dysfunction and failure. For progression of the fibrotic processes, myofibroblasts originating from several sources, including quiescent tissue fibroblasts and epithelial or endothelial cells, have been recognized as the responsible cells upregulating the expression of α-SMA and increasing the production of extracellular matrix proteins, including various types of collagens [75]. TGF-β plays a central role in the fibrogenesis by modulating the fibroblast phenotype and function, inducing myofibroblast transdifferentiation and promoting matrix accumulation [13]. In addition, pro-inflammatory cytokines released by macrophages and potentially by neutrophils, mast cells and B-lymphocytes, e.g., TNFα and IL-1, may contribute to the regulation of lung fibrosis [76]. In our study, the concentration of TGF-β1 in the lung homogenate was elevated after 14 days of silica exposure, and persisted significantly higher than in controls until the end of the experiment. This result is in agreement with other experimental studies, where TGF-β1 increased in BALF due to 28 days of silica exposure [23], in the lung after 8 weeks [22], or after 56 days of silica exposure [53], respectively. Direct evidence of collagen deposition in this study was performed immunohistochemically by staining with Sirius red. We found that the content of collagen in the walls of bronchioles and pulmonary vessels significantly increased after 14 days of silica exposure, which was further confirmed by slightly elevated concentrations of hydroxyproline, a major component of collagen, in the lung homogenate. Similar to our results, a higher collagen content and hydroxyproline concentrations in the silica-instilled lung were demonstrated by other authors [23], while Abdelaziz et al. [22] showed an obvious interstitial inflammation, peribronchial fibrosis, and an elevation in the inter-alveolar septa and blood vessel thickness compared to healthy controls. As a marker of myofibroblast formation, α-SMA was investigated immunohistochemically using a specific antibody. We found that the mass of the smooth muscle in the walls of bronchioles and pulmonary vessels indicated by α-SMA significantly increased in the silica-instilled animals after 14 days, and the values elevated in time. An increase in α-SMA expression or lung content was also observed in other experimental models of silicosis [23,27,77]. Nevertheless, silica-induced changes may lead to vascular remodeling [6], as we could see in our study, as increased collagen and connective tissue mass in the wall of pulmonary vessels. Inhaled particulate matter might be transported into the vessels directly by the blood or lymph by migrating phagocytes, or the endothelial dysfunction and deleterious effects to the cardiovascular system may be mediated indirectly by silica-induced inflammatory mediators [78,79]. It is presumed that systemic vascular dysfunction correlates with a degree of pulmonary inflammation and systemic inflammation associated with the elevation of systemic pro-inflammatory cytokines (IL-6, TNFα) [80,81].

When a time exposure was evaluated, an increase in some inflammatory markers, e.g., NLRP3, TNFα, IL-1, was pronounced after 14 days of the experiment, suggesting predominant inflammatory changes. However, the early phase of silica-induced injury was also associated with significant fibrotic changes expressed by elevations in hydroxyproline and TGF-β1 levels, and a significant fibrotization of bronchioles and pulmonary vessels verified immunohistochemically, indicating that inflammatory and fibrotic changes develop simultaneously. Later, an increase in the counts of BALF leukocytes gradually boosted and was linked not only with an increase in the W/D ratio, but also with more serious fibrotic changes after 28 days of the experiment.

In this study, the silica-instilled rats were treated by intraperitoneal EGCG. EGCG exerts a wide variety of properties that may be useful in the treatment of pulmonary silicosis; however, there has been insufficient information available about the effects of EGCG in lung silicosis. In our experiments, the administration of EGCG twice a week decreased a total count of cells as well as counts of neutrophils, eosinophils and lymphocytes in the BALF that was associated with their reciprocal increases in the peripheral blood. As markers of alleviated inflammation, we could consider a slight decrease in the W/D ratio after 28 days, significant decreases in the lung concentrations of TNFα after 14 and 28 days, and declines of IL-6 and CXCL1 after 28 days. Concentrations of SLC26A4, NRLP3, NF-κB, and IL-1β decreased after 14 days, but there was no difference or even a slight increase after 28 days. Similarly, inconsistent effects were observed in oxidant/antioxidant markers, while EGCG increased the concentration of catalase and slightly decreased 3-nitrotyrosine, but simultaneously decreased the concentrations of TAC and SOD. Expression of Nrf2 slightly increased after 28 days; however, HO-1 did not change, and after a significant increase after 14 days, NQO1 significantly decreased after 28 days. We can hypothesize that these inconsistent results of several inflammatory and oxidant/antioxidant markers may result from a concentration-dependent character of biological effects of EGCG. While low concentrations exert a predominantly antioxidant effect, higher concentrations may be pro-oxidative [82]. Thus, according to the actual concentration of EGCG in the tissues, we may presume antioxidant but also some pro-oxidant effects simultaneously. Similarly, although EGCG increased both pro-apoptotic Bax protein and anti-apoptotic protein Bcl-2 after 14 days, the situation changed after 28 days when EGCG increased Bcl-2 and decreased Bax. This resulted into a significant decrease of the Bax/Bcl-2 ratio, which may suggest a decline in the lung cell apoptosis; however, further analyses, including a TUNEL investigation or caspase-3 analysis, should be performed to prove our findings. Finally, EGCG significantly decreased the accumulation of collagen and connective tissue mass in the walls of bronchioles and pulmonary vessels compared to the silica-instilled non-treated animals, which was associated with slightly lower concentrations of hydroxyproline and TGF-β1, demonstrating an anti-fibrotic potential of EGCG in this model of lung silicosis. 

The favorable therapeutic action of EGCG was also presented in other animal models of lung fibrosis. For instance, in bleomycin-induced fibrosis EGCG prevented a generation of ROS, elevated levels of antioxidants including Nrf2 activity, reduced a lung edema, decreased levels of NF-κB, TNFα, IL-1β and myeloperoxidase activity, decreased a content of hydroxyproline, decreased an expression of TGF-β1 and α-SMA, and alleviated a lung injury [41,42,43]. In an irradiation-induced model of fibrosis, EGCG decreased a severity of lung histological changes, lowered levels of malonyldialdehyde (a marker of lipid peroxidation) in the lungs and serum levels of TGF-β1, IL-6, IL-10, and TNFα, activated Nrf2 and associated antioxidant enzymes HO-1 and NQO-1, enhanced activity of SOD, and lowered collagen deposition and (myo)fibroblast proliferation [44]. Similarly, a green tea extract ameliorated paraquat-induced [46] and cyclophosphamide-induced pulmonary fibrosis [45]. 

Apart from our study, the only study evaluating effects of EGCG in the animal model of pulmonary silicosis is the recent work by Yao et al. [47]. These authors used a similar model of silicosis to ours, i.e., a silicosis model was induced in Sprague-Dawley rats by intratracheal instillation of silica suspension (1 mL, 50 mg/mL), and the results were estimated after 14 or 28 days of the treatment, respectively. The authors compared the effects of EGCG (50 mg/kg) and encapsulated EGCG in poly(n-butylcyanoacrylate) nanoparticles (EGCG/PBCA-NP, containing 50 mg/kg of EGCG) delivered daily by a gastrointestinal administration which was initiated 2 days after the silica instillation. The encapsulation enhanced bioavailability of EGCG that resulted in superior therapeutic effects compared to naked EGCG. Treatment with EGCG prevented a silica-induced increase in the lung index (i.e., lung weight/body weight) and decreased hydroxyproline content in the lung after 14 and 28 days, while encapsulated EGCG showed more potent action. The limited effect of EGCG was demonstrated on collagen accumulation, differentiation of fibroblasts to myofibroblasts, and overexpression of α-SMA in the lung, while the effect of encapsulated EGCG was more significant [47]. In our study, an intraperitoneal administration of EGCG was chosen to avoid a significant degradation in the gastrointestinal system, which causes only about 1% bioavailability after oral administration of EGCG [83]. This is probably the reason why some results from our study showed more potent effects of the treatment than in the study by Yao et al. [47], despite the higher doses and daily administration which were used in their study. On the other hand, a systemic route of administration may limit a wider clinical use of EGCG. However, advanced EGCG delivery forms may represent a favorable way for how to solve this problem [40].

Of course, we are aware of some of the limitations of our study. While the dynamics of progression of the lung silicosis in patients inhaling silica particles gradually develops over years, distribution of the silica particles after artificial instillation in the laboratory animals is different. However, as published in previous studies [2,6,56], instilled silica causes a rather homogenic local response, and the silica-induced inflammatory and fibrotic changes in the lungs of laboratory animals are very similar to the changes observed in patients suffering from lung silicosis. Even a single intratracheal quartz instillation in animals may cause serious inflammatory changes after 52 weeks. However, it may also result into higher incidences of adenoma and adenocarcinoma after 96 weeks, suggesting a close relation between silica-induced chronic inflammation and lung cancer [51]. In addition, the deleterious effects of silica seem to be strongly dependent on time relations, the route of silica administration, and animal species/strains used for developing a silicosis model. This may lead to some discrepancies among the studies. For instance, Porter et al. [56] demonstrated early changes after 5 days of the daily silica exposure, and the severity increased linearly with continued exposure to silica, while more significant changes, e.g., in counts of neutrophils and alveolar macrophages in BALF, concentrations of IL-1 and TNFα, or more serious fibrotic changes were observed after 41 days of silica exposure. The differences between our results and the other studies could be additionally explained by other methods of silica instillation (i.e., orotracheal instillation of silica in our study), as well as by possible strain differences in rats in the inflammatory response to silica, as Sprague-Dawley rats may show a stronger inflammatory response to silica than Wistar-Hannover rats [84]. An additional objection to our study may originate from the sole use of male animals. This has resulted from the findings of other authors that female animals are more susceptible to the instillation of fibrotizing agents, including silica, that may result in more severe inflammatory and fibrotic changes as well as a higher mortality rate [85,86]. Therefore, male animals are commonly used for preparing animal models of various forms of lung fibrosis.

## 4. Materials and Methods

### 4.1. Silica

For intratracheal instillation, silica particles (SiO_2_ powder, 1.5 micron, 99.9%; Alfa Aesar, ThermoFisher, Germany) were used. Before instillation, silica powder was sterilized in the oven at 200 °C for 2 h to avoid a bacterial contamination. Subsequently, the required amount of silica was weighed and suspended in a sterile saline at the concentration of 50 mg/mL. Before instillation, the suspension was placed in a sonicator for 5 min and mixed again.

### 4.2. Animals

Adult male Wistar rats of the mean body weight (b.w.) of 200–250 g were supplied by a certified animal breeding station (VELAZ, Prague 6, Czech Republic). Animals were kept in a certified faculty animal house where they underwent 7-days quarantine and acclimation, with available food and water ad libitum. This study was approved by the National Veterinary Board of Slovakia and the Ethical Committee of Jessenius Faculty of Medicine in Martin, Comenius University in Bratislava.

### 4.3. General Design of Experiments

Animals (n = 48 in total) were divided into three groups (Control, Model, EGCG), which were then each subdivided into 2 subgroups and euthanized after 14 days (i.e., for evaluation of inflammatory changes) or after 28 days of the therapy administration (i.e., for evaluation of early fibrotic changes). The model of lung silicosis was induced during a short inhalation anesthesia (4% isoflurane) by a single transoral intratracheal instillation of silica suspension (1 mL/animal) (Model groups), while the control animals (Control groups) received an equivalent volume of sterile saline. Instillation of silica/saline was performed on an angled board by hooking the front teeth while positioning the tongue laterally, which enabled the instillation of the silica suspension/saline by micropippetor into the trachea through opened vocal cords during inspiration [modified according to [87,88]].

**Figure 6:**
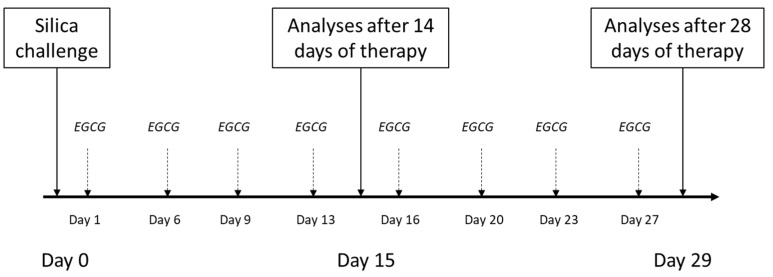
Schematic study protocol for induction of the silicosis and delivery of the treatment. Silica particles suspended in saline were instilled orotracheally at day 0. Treatment with intraperitoneal EGCG at a dose of 20 mg/kg b.w. was initiated the next day after silica instillation (day 1) and was given twice a week. The analyses were performed after 14 days and 28 days of the treatment administration.

In half of the silica-instilled animals, the treatment with intraperitoneal EGCG (Epigallocatechin gallate, PHR1333, Sigma-Aldrich, St. Louis, MO, USA) was initiated a next day after the silica instillation, and was given twice a week at a dose of 20 mg/kg b.w. [according to [89]], dissolved in a sterile saline up to volume of 1 mL/kg (EGCG groups), while the other animals received an equivalent volume of sterile saline (Model groups). Animals were euthanized after 14 days (Control14, Model14 and EGCG14 subgroups, each of n = 8) or 28 days (Control28, Model28 and EGCG28 subgroups, each of n = 8) after the treatment onset by an overdosing of anesthetics (Zoletil, Virbac, Carros, France). Schematic protocol of the study is provided in Figure 6.

### 4.4. Total and Differential Counts of Leukocytes in the Blood and in the BALF

Samples of blood were taken by a direct puncture of the heart. Total count of leukocytes and absolute counts of individual types of leukocytes (×10^3^/μL) were determined by a veterinary hematologic analyzer (Sysmex XT-2000iV, Kobe, Japan). Differential count of leukocytes was also estimated microscopically after staining by May-Grünwald/Giemsa-Romanowski and expressed in percents (%). 

The left lung was lavaged with saline (0.9% NaCl, 2 × 10 mL/kg b.w.). The total count of cells in the BALF was measured by a cell analyzer (Countess, Thermo Fisher Scientific, Waltham, MA, USA). Then, the BALF was centrifuged at 1500 rpm for 15 min. Differential count of cells in the BALF sediment was evaluated microscopically after staining by May-Grünwald/Giemsa-Romanowski and expressed in percents (%). From these data, the absolute values of individual leukocyte types were calculated (expressed as × 10^3^/mL).

### 4.5. Analysis of Markers of Inflammation, Oxidative Stress, and Fibrosis by Enzyme Immunosorbent Analysis (ELISA) Methods

Concentrations of inflammation markers, oxidative stress, and fibrosis were determined in 10% (weight/volume) homogenates of the right lung tissue, which were prepared using 0.1 M ice-cold phosphate buffer (PBS, pH 7.4) and homogenized 5-times for 25 s at 1200 rpm (Polytron homogenizer PT 1200 E, Kinematica AG, Malters, Switzerland). Homogenates were then frozen 3 times and centrifuged (12,000 rpm, 15 min, 4 °C). Final supernatants were then stored at −70 °C until the analysis was performed. 

The ELISA methods was used for measurements of concentrations of below-listed parameters: TNFα (ab236712, Abcam, Cambridge, UK), IL-1β (ab255730, Abcam, Cambridge, UK), IL-6 (ab234570, Abcam, Cambridge, UK); HO-1 (ER1041, FineTest, Wuhan Fine Biotech Co., Ltd., Wuhan, China), hydroxyproline (MAK357, Sigma Aldrich, USA), TGF-β1 (ER1378, FineTest, Wuhan Fine Biotech Co., Ltd., Wuhan, China), NLRP3 (ER1965, FineTest, Wuhan Fine Biotech Co., Ltd., Wuhan, China); catalase, SOD, TAC, and 3-nitrotyrosine (Catalase Activity assay kit, Superoxide Dismutase Activity Assay, Total antioxidant capacity assay, Nitrotyrosine assay, OxiSelect, Cell Biolabs, Inc., San Diego, CA, USA). The manufacturers’ protocols were strictly followed during the measurements and all ELISA analyses were performed in duplicates. 

### 4.6. Analysis of Markers of Inflammation, Oxidative Stress, and Fibrosis by Polymerase Chain Reaction (PCR) Methods

PCR analysis was performed according to our previous study [90]. Briefly, strips of the right lung tissue were put into RNAlater^®^ solution (Sigma-Aldrich, Burlington, MA, USA), stabilizing and protecting ribonucleic acid (RNA), stored overnight at 4 °C, and then the samples were frozen at −80 °C. Total RNA from the sample was extracted using RNeasy kit (Qiagen, Inc., Hilden, Germany) with the β-mercaptoethanol according to the manufacturer’s protocol. RNA quality and concentration were at first measured by Nanodrop spectrophotometer (Thermo Fisher Scientific, Inc., Waltham, MA, USA). All mRNA was detected using SYBR Green+UNG Master Mix (Qiagen, Inc., Hilden, Germany) in real-time (RT) PCR detection system (iCycler, Bio-Rad, model IQ5, Hercules, CA, USA). The first step was heating for 2 min at 50 °C (due to the presence of uracil-N-glycosylase, UNG), after enzyme activation at 95 °C for 15 min, and the amplification protocol is composed of denaturation for 15 s at 94 °C, annealing for 30 s at 55 °C and extension for 30 s at 72 °C for 40 cycles. Fold changes in the expression levels were calculated using the 2^−∆∆Ct^ method and normalized using GAPDH as an endogenous control. All qPCR analyses were performed in triplicates. The gene-specific primers for rats used in this study are listed in Table 4.

### 4.7. Determination of Wet-Dry (W/D) Lung Weight Ratio

Strips of the right upper lung lobe were weighed and dried at 60 °C for 24 h and then the W/D ratio was determined. The higher value indicates the higher accumulation of liquid in the lung. 

### 4.8. Immunohistochemical Analyses of Fibrotic Changes in the Lung

The right lung was washed with saline and stored in 4% formaldehyde. The presence of collagen (by Sirius red staining) and smooth muscle mass (detection of smooth muscle actin, SMA) in the walls of bronchioles and vessels of the right lung was verified immunohistochemically by a qualified histologist. The lung tissue was dehydrated through a series of graded ethanol baths, infiltrated with paraffin and cut into 4 µm thick sections.

Detection of collagen by Sirius red: after deparaffinization the slides were firstly stained with Weigert’s hematoxylin for visualization of the cell nuclei. After washing in running tap water (10 min), the slides were stained in Picro-Sirius red solution (Millipore Sigma, St. Louis, MO, USA) for 1 h. After washing twice in acidic distilled water, and dehydration in 100% ethanol, the slides were mounted in Entellan (Millipore Sigma, Burlington, MA, USA). The result of the staining was red collagen fibers on a pale yellow background. The slides were viewed with an Olympus BX43 microscope (Olympus, Tokyo, Japan). The image capture and airway wall thickness measurement were performed with Quick Photo Micro software, version 3.2 (Olympus, Tokyo, Japan). 

Detection of smooth muscle mass by SMA: after deparaffinization, revitalization and rehydration, the tissue slides were treated with 3% H_2_O_2_ solution for 10 min for blocking endogenous peroxidases. Washing with Tris buffer was used after each handling step. The sections were incubated with the primary rabbit polyclonal smooth muscle actin (SMA; 1:300, Cell Signaling Technology, Danvers, MA, USA) for 30 min at room temperature. The slides were then incubated by sequential 10 min incubation with LSAB2 System-HRP for use on rat specimens (Dako, Glostrup, Denmark), which detects primary mouse and rabbit antibodies. The sections were then counterstained with Mayer’s hematoxylin (Himedia Laboratories, Maharashtra, India) and mounted with an Entellan (Merck, USA). The sections were viewed with an Olympus BX43 microscope (Olympus, Japan) equipped with photo camera Canon E0S 2000D. The Quick Photo Micro program, version 3.2 (Olympus, Rahway, NJ, Japan) was used to image capture the sections and measure the thickness of the smooth muscle layer in the bronchial wall and the *tunica media* in the blood vessel wall (expressed by dark brown cytoplasm of SMA-positive cells). 

### 4.9. Statistical Analysis

For analysis of the data, statistical package GraphPad (San Diego, CA, USA) was used. Differences among the groups were analyzed by non-parametric Mann–Whitney test. A value of *p* < 0.05 was considered statistically significant. Data are expressed as means ± SD.

## 5. Conclusions

Due to a complex pathophysiology, treatment of pulmonary silicosis is extremely difficult. Nevertheless, recent studies indicate that herbal compounds, including EGCG, may slow down the development of lung inflammation and fibrosis. Due to the fact that until now, there was insufficient data on EGCG delivery in animal models of silicosis, our study aimed to evaluate several markers of inflammation, oxidative stress, apoptosis, and fibrosis to estimate whether EGCG may mitigate early inflammatory and fibrotic changes in the lungs of silica-instilled rats. Our results showed some potential for EGCG to alleviate inflammation and a trend to decrease oxidative stress-induced changes including apoptosis, and a prevention of fibrotic changes in the bronchioles and pulmonary vessels. However, further investigations should be undertaken to elucidate the effects of EGCG in the lung silicosis model in more detail. In addition, positive and eventual adverse effects of this herbal compound should be carefully studied before any preventive use or therapy with EGCG may be recommended. 

## Figures and Tables

**Table 1:** Counts of leukocytes in the blood of saline-instilled animals (Control), silica-instilled non-treated animals (Model), and EGCG-treated silica-instilled animals (EGCG) after 14 days (14d) or 28 days (28d) of the treatment delivery.

	Control14d	Model14d	EGCG14d	Control28d	Model28d	EGCG28d
Total count of leukocytes in the blood (×10^3^/µL)						
	3.7 ± 1.2	5.4 ± 1.2 *	6.4 ± 1.7	3.7 ± 1.1	5.0 ± 1.2	6.5 ± 0.8 ^&^
Absolute counts of leukocyte types in the blood (×10^3^/µL)						
Neu	0.64 ± 0.17	0.94 ± 0.38	2.20 ± 0.65 ++	0.52 ± 0.17	0.76 ± 0.29	2.02 ± 0.60 ^&&&^
Lym	2.90 ± 1.10	4.26 ± 0.84 *	4.50 ± 1.50	3.00 ± 0.93	3.96 ± 1.00	4.10 ± 0.87
Mon	0.08 ± 0.05	0.12 ± 0.06	0.20 ± 0.05	0.09 ± 0.04	0.16 ± 0.06 #	0.26 ± 0.05 ^&&^
Eos	0.05 ± 0.06	0.05 ± 0.03	0.07 ± 0.03	0.05 ± 0.04	0.07 ± 0.05	0.06 ± 0.03
Relative counts of leukocyte types in the blood (%)						
Neu	18.4 ± 4.9	17.0 ± 4.2	32.3 ± 9.3 +	14.6 ± 3.7	15.4 ± 4.6	31.6 ± 9.2 ^&&&^
Lym	78.3 ± 4.7	79.8 ± 5.0	63.5 ± 10.5 ++	81.7 ± 3.8	79.9 ± 5.6	63.5 ± 9.6 ^&&^
Mon	2.0 ± 1.0	2.3 ± 0.8	3.0 ± 1.0	2.4 ± 1.1	3.2 ± 1.0	4.0 ± 0.5
Eos	1.3 ± 1.3	0.9 ± 0.5	1.1 ± 0.6	1.3 ± 1.2	1.4 ± 0.7	0.8 ± 0.4 ^&^

Abbreviations: Neu: neutrophils, Lym: lymphocytes, Mon: monocytes, Eos: eosinophils. For statistical differences among the groups: * *p* < 0.05 for Model14d vs. Control14d; + *p* < 0.05 and ++ *p* < 0.01 for EGCG14d vs. Model14d; # *p* < 0.05 for Model28d vs. Control28d; ^&^ *p* < 0.05, ^&&^ *p* < 0.01 and ^&&&^ *p* < 0.001 for EGCG28d vs. Model28d.

**Table 2:** Counts of leukocytes in the BALF of saline-instilled animals (Control), silica-instilled non-treated animals (Model), and EGCG-treated silica-instilled animals (EGCG) after 14 days (14d) or 28 days (28d) of the treatment delivery.

	Control14d	Model14d	EGCG14d	Control28d	Model28d	EGCG28d
Total count of leukocytes in the BALF (×10^3^/mL)						
	70.9 ± 52.1	97.5 ± 41.0	95.0 ± 35.8	121.3 ± 44.9	135.7 ± 68.5	96.3 ± 36.6
Absolute counts of leukocyte types in the BALF (×10^3^/mL)						
Mac	97.7 ± 41.5	86.1 ± 44.5	89.1 ± 35.1	142.1 ± 101.5	108.3 ± 61.0	90.6 ± 38.5
Neu	2.3 ± 1.7	12.4 ± 4.7 ***	3.7 ± 2.0 +++	3.8 ± 1.5	20.8 ± 10.3 ###	9.3 ± 3.1 ^&^
Lym	1.0 ± 0.6	2.1 ± 1.7	1.5 ± 1.2	1.0 ± 0.7	4.7 ± 1.9 ##	1.8 ± 1.2 ^&^
Eos	0.3 ± 0.3	0.7 ± 0.3 *	0.7 ± 0.3	0.2 ± 0.2	1.7 ± 1.5 #	0.8 ± 0.8
Relative counts of leukocyte types in the BALF (%)						
Mac	96.7 ± 1.1	82.6 ± 6.5 ***	92.9 ± 4.4 ++	96.1 ± 1.1	77.0 ± 8.9 ###	87.2 ± 5.8 ^&^
Neu	2.1 ± 0.9	14.7 ± 6.9 ***	4.6 ± 3.5 ++	3.1 ± 1.2	17.4 ± 6.9 ###	10.2 ± 5.2 ^&^
Lym	0.9 ± 0.3	1.9 ± 1.1 *	1.7 ± 1.3	0.7 ± 0.2	3.9 ± 1.8 ###	1.8 ± 1.2 ^&^
Eos	0.2 ± 0.3	0.8 ± 0.2 **	0.8 ± 0.4	0.2 ± 0.2	1.8 ± 1.4 ###	0.8 ± 0.6

Abbreviations: Mac: macrophages, Neu: neutrophils, Lym: lymphocytes, Eos: eosinophils. For statistical differences among the groups: * *p* < 0.05, ** *p* < 0.01 and *** *p* < 0.001 for Model14d vs. Control14d; ++ *p* < 0.01 and +++ *p* < 0.001 for EGCG14d vs. Model14d; # *p* < 0.05, ## *p* < 0.01 and ### *p* < 0.001 for Model28d vs. Control28d; ^&^ *p* < 0.05 for EGCG28d vs. Model28d.

**Table 3:** Summarization of effects of EGCG treatment (EGCG14d and EGCG28d groups) on different markers of inflammation, oxidative stress, apoptosis, and fibrosis after 14 days or 28 days of the experiment compared to silica-instilled and EGCG non-treated animals (Model14d and Model28d groups).

Markers	After 14 Days	After 28 Days
Inflammation		
Total blood leukocytes	↑ NS	↑ *p* < 0.05
Absolute blood neutrophils	↑ *p* < 0.01	↑ *p* < 0.001
Absolute blood lymphocytes	↑ NS	↑ NS
Absolute blood monocytes	↑ NS	↑ *p* < 0.01
Absolute blood eosinophils	↑ NS	↓ NS
Total BALF leukocytes	↓ NS	↓ NS
Absolute BALF macrophages	↑ NS	↓ NS
Absolute BALF neutrophils	↓ *p* < 0.001	↓ *p* < 0.05
Absolute BALF lymphocytes	↓ NS	↓ *p* < 0.05
Absolute BALF eosinophils	-	↓ NS
W/D ratio	-	↓ NS
NLRP3	↓ *p* < 0.05	-
NF-κB	↓ NS	↑ NS
TNFα	↓ *p* < 0.001	↓ *p* < 0.05
IL-1β	↓ NS	↑ NS
IL-6	-	↓ *p* < 0.01
CXCL1	-	↓ *p* < 0.01
SLC26A4	↓ *p* < 0.05	↓ NS
Oxidative stress		
3-nitrotyrosine	↓ NS	↓ NS
TAC	↓ NS	↓ NS
SOD	↓ *p* < 0.01	↓ *p* < 0.01
Catalase	-	↑ *p* < 0.05
Nrf2	-	↑ NS
HO-1	-	-
NQO1	↑ *p* < 0.01	↓ *p* < 0.05
Apoptosis		
Bax/Bcl2	-	↓ *p* < 0.05
**Fibrosis**		
Hydroxyproline	↓ NS	↓ NS
TGF-β1	↓ NS	↓ NS
Collagen—bronchioles (IHI)	↓ *p* < 0.01	↓ *p* < 0.01
Collagen—vessels (IHI)	↓ *p* < 0.01	↓ *p* < 0.01
α-SMA—bronchioles (IHI)	↓ *p* < 0.01	↓ *p* < 0.01
α-SMA—vessels (IHI)	↓ *p* < 0.01	↓ *p* < 0.05

Abbreviations and marks: NS: non-significant statistical difference for EGCG-treated vs. non-treated groups; ↑: increase; ↓: decrease; -: no change; BALF: bronchoalveolar lavage fluid; W/D ratio: wet/dry lung weight ratio; NLRP3: nucleotide-binding and oligomerization domain-like receptor; NF-κB: nuclear factor-kappa B; TNFα: tumor necrosis factor alpha; IL-1β: interleukin-1beta; IL-6: interleukin-6; CXCL1: chemokine (C-X-C motif) ligand 1; SLC26A4: solute carrier family 26, member 4; TAC: total antioxidant capacity; SOD: superoxide dismutase; Nrf2: nuclear factor erythroid-derived 2-like factor 2; HO-1: heme oxygenase; NQO-1: NAD(P)H:quinone oxidoreductase-1; Bcl2: B-cell lymphoma 2 protein; Bax: Bcl-2 associated X-protein; TGF-β1: transforming growth factor-beta 1; IHI: immunohistochemical investigation.

**Table 4:** The primer sequences amplified used for RT-PCR.

Primers	Forward	Reverse
Nrf2	TCTGACTCCGGCATTTCACT	TGTTGGCTGTGCTTTAGGTC
NQO-1	CATCATTTGGGCAAGTCC	ACAGCCGTGGCAGAACTA
Bcl-2	GGGATGACTTCTCTCGTCGC	AGAGCGATGTTGTCCACCAG
Bax	AGGACGCATCCACCAAGAAG	GGGGGTCCCGAAGTAGGAAA
CXCL1	GGCAGGGATTCACTTCAAGAACATC	AGTGTGGCTATGACTTCGGTTTGG
NF-κB	TCTGACTCCGGCATTTCACT	TGTTGGCTGTGCTTTAGGTC
SLC26A4	GGGCAACCAAGAACGGGATTATAAG	TCTGGCTCTTCGACATCTTCATCAG
GAPDH	GGCACAGTCAAGGCTGAGAATG	ATGGTGGTGAAGACGCCAGTA

Abbreviations: Nrf2: nuclear factor erythroid-derived 2-like factor 2; NQO-1: NAD(P)H:quinone oxidoreductase-1; Bcl-2: B-cell lymphoma 2 protein; BAX: Bcl-2 associated X-protein; CXCL1: chemokine (C-X-C motif) ligand 1; NF-κB: nuclear factor-kappa B; SLC26A4: solute carrier family 26, member 4; GAPDH: glyceraldehyde 3-phosphate dehydrogenase.

## Data Availability

Data is contained within the article.
